# Establishing the epidemiology of respiratory viral infections using “A NOVEL POINT-OF-CARE MULTIANALYTE ANTIGEN DETECTION TEST mariPOC” during the season 2013-2014

**DOI:** 10.1186/1471-2334-14-S7-O33

**Published:** 2014-10-15

**Authors:** Angelica Vişan, Anca Drăgănescu, Anuța Bilaşco, Cristina Negulescu, Camelia Kouris, Ruxandra Măntescu, Gheorghiță Jugulete, Mădălina Merişescu, Endis Osman, Cornelia Dogaru, Diana Slavu, Monica Luminos

**Affiliations:** 1 National Institute for Infectious Diseases "Prof. Dr. Matei Balş", Bucharest, Romania; 2 Carol Davila University of Medicine and Pharmacy, Bucharest, Romania

## Background

Rapid etiological diagnosis has a very important role in the clinical management of respiratory viral infections. Using a multianalyte point-of-care detection system, based on a fully automated immunoassay method, we can detect 8 respiratory viruses (influenza A and B viruses, parainfluenza 1, 2 and 3 viruses, respiratory syncytial virus, human metapneumovirus and adenovirus) and the presence of *Streptococcus pneumoniae* from a single nasopharyngeal swab or aspirate. Objectives: To evaluate the incidence of respiratory viral infections in the pediatric department of the National Institute for Infectious Diseases "Prof. Dr. Matei Balş"during 1 November 2013 – 1 June 2014.

## Methods

The collected samples from children with respiratory tract symptoms were analyzed by mariPOC (the novel multianalyte point-of-care antigen detection test). Positive samples were then studied in terms of clinical manifestations, complications, signs of bacterial co-infection, antiviral and antibiotic administration and days of hospitalization.

## Results

We tested approximately 600 samples, out of which 50% were positive for at least one virus. The most frequent infection was influenza A, which accounted for 55% of the positive samples. Other frequent viruses found were respiratory syncytial virus in 27% of cases, human metapneumovirus in 6.2%. We found viral co-infections in 8.9% of cases, out of which the most frequent association was influenza A virus with respiratory syncytial virus.

## Conclusion

The mariPOC antigen detection test provides a very useful and rapid pathogen specific diagnosis of respiratory infections, having a high specificity for the most important viruses. Using this method we found that influenza A virus was the most frequent viral infection in children during 2013-2014 winter-spring season, but also that viral co-infections are an important etiology of respiratory symptoms in children.

